# Reelin Signaling Controls the Preference for Social Novelty in Zebrafish

**DOI:** 10.3389/fnbeh.2019.00214

**Published:** 2019-09-19

**Authors:** Elisa Dalla Vecchia, Vincenzo Di Donato, Andrew M. J. Young, Filippo Del Bene, William H. J. Norton

**Affiliations:** ^1^Department of Neuroscience, Psychology and Behaviour, University of Leicester, Leicester, United Kingdom; ^2^Institut Curie, Paris, France; ^3^ZeClinics SL, Institute for Health Science Research Germans Trias i Pujol (IGTP), Barcelona, Spain

**Keywords:** zebrafish, Reelin, behavior, autism, neurochemistry, social behavior, neuroscience, 5-HT

## Abstract

Reelin (Reln) is an extracellular glycoprotein that is important for brain patterning. During development Reln coordinates the radial migration of postmitotic cortical neurons, cerebellar and hippocampal neurons, whereas it promotes dendrite maturation, synaptogenesis, synaptic transmission, plasticity and neurotransmitter release in the postnatal and adult brain. Genetic studies of human patients have demonstrated association between the RELN locus and autism spectrum disorder, schizophrenia, bipolar disorder, and Alzheimer’s disease. In this study we have characterized the behavioral phenotype of *reelin* (*reln*) mutant zebrafish, as well as two canonical signaling pathway targets *DAB adaptor protein 1a* (*dab1a*) and the *very low density lipoprotein receptor* (*vldlr*). Zebrafish *reln^–/–^* mutants display a selective reduction in preference for social novelty that is not observed in *dab1a^–/–^* or *vldlr^–/–^* mutant lines. They also exhibit an increase in 5-HT signaling in the hindbrain that parallels but does not underpin the alteration in social preference. These results suggest that zebrafish *reln^–/–^* mutants can be used to model some aspects of human diseases in which changes to Reln signaling alter social behavior.

## Introduction

Reelin (Reln) is an extracellular glycoprotein that is important for brain patterning and synaptogenesis. During mammalian development Reln is secreted by Cajal-Retzius (C-R) neurons in cerebral cortex and granule cells in the external granule cell layer of the cerebellum (Pesold et al., 1998; Lee and D’Arcangelo, 2016). In the adult brain Reln is expressed by γ-aminobutyric acid (GABA)-positive interneurons in the cortex and hippocampus, and glutamatergic granule cells in the cerebellum (Pesold et al., 1998). The mammalian neocortex develops in an inside-out manner in which late-born neurons migrate past existing neurons to form a laminar structure (Rakic, 2009). Reln coordinates the radial migration of postmitotic cortical neurons, cerebellar and hippocampal neurons (D’Arcangelo, 2014). Reeler mice that lack Reln function display a disorganization of cortical lamination and cerebellar hypoplasia caused by the failure of Purkinje neurons to migrate (Goffinet, 1983; Miyata et al., 1997). Similar abnormalities have been identified in human patients with homozygous null mutations in *RELN*, who display lissencephaly and cerebellar hypoplasia (Hong et al., 2000). In the mature brain, RELN promotes dendrite maturation, synaptogenesis, synaptic transmission, plasticity and the release of neurotransmitters (D’Arcangelo, 2005; Herz and Chen, 2006; Levenson et al., 2008; Förster et al., 2010; Levy et al., 2014).

Reln signaling is transduced by several downstream pathways including two low-density lipoprotein receptor family members: apolipoprotein E receptor 2 (ApoER2, also called low-density lipoprotein receptor-related protein 8, LRP8) and the very low density lipoprotein receptor (Vldlr) (Trommsdorff et al., 1999). ApoER2 and Vldlr are expressed on the membrane of target neurons. Their activation leads to internalization of Reln in endocytic vesicles, phosphorylation of the DAB adapter protein 1 (Dab1; Howell et al., 1997; Sheldon et al., 1997) and activation of Src/Fyn kinases as part of canonical Reln signaling (D’Arcangelo, 2006; Bock and May, 2016). ApoER2 and VLDLR have partially redundant functions and double *Apoer2/Vldlr* knock-out mice exhibit a *reeler*-like neuroanatomical phenotype (Trommsdorff et al., 1999). However, they also play specific roles in neuronal migration. ApoER2 is mostly expressed in hippocampal and cortical neurons and single *Apoer2* knock-out mice show defects in forebrain structures. *Vldlr* is mostly expressed in the Purkinje cells of the cerebellum, and these neurons fail to assemble in a tight layer in *Vldlr* knock-out mice (Trommsdorff et al., 1999). Other signaling pathways regulated by Reln include CrK/Rap1 (neuronal migration and lamination) and the P13K/Akt/mTOR pathway (dendrite and spine development). Activity at NMDA receptors and the MEK/Erk1/2 pathway enhances the expression of synaptic plasticity and learning genes (Chen et al., 2010).

Animal studies further support a link between decreased Reln signaling and behavioral abnormalities. Both homozygous and heterozygous *reeler* mice exhibit behavioral and cognitive defects (Tueting et al., 1999; Costa, 2002; Qiu et al., 2006). *Reln*^+/–^ mice show impaired prepulse inhibition, a sensory motor gaiting phenotype associated with schizophrenia (Tueting et al., 1999; Barr et al., 2008; Kutiyanawalla et al., 2011; Teixeira et al., 2011) although not all studies agree (Qiu et al., 2006; Teixeira et al., 2011). *Reln*^+/–^ mice also exhibit neophobia in the elevated plus maze, a decrease in cortical dendritic spine density and defects in hippocampus-based learning (Qiu et al., 2006), long-term potentiation and long-term depression (Iafrati et al., 2013). Cross-modal prepulse inhibition is increased in *Apoer2^–/–^* knock-out mice and decreased in *Vldlr^–/–^* (Weeber et al., 2002); and *Dab1*-conditional knockout mice exhibit hyperactivity and deficits in anxiety and working memory (Imai et al., 2016).

Abnormal Reln signaling has also been linked to several human psychiatric disorders. Genetic studies have demonstrated an association between the RELN locus and autism spectrum disorder (ASD; Wang et al., 2014), schizophrenia (Ovadia and Shifman, 2011; Li et al., 2015), bipolar disorder (Ovadia and Shifman, 2011), and Alzheimer’s disease (Bufill et al., 2013). In humans, RELN is located at chromosome 7q22, the peak region of linkage and first autism susceptibility locus (AUTS1). Linkage and epidemiologic studies have yielded both positive and negative findings as to whether RELN plays a major role in ASD susceptibility (Persico et al., 2001; Krebs et al., 2002; Zhang et al., 2002; Bonora et al., 2003; Li et al., 2004; Dutta et al., 2006, 2008; Serajee et al., 2006; Li et al., 2008; He et al., 2011). Meta-analysis of several studies has identified a single nucleotide polymorphism that segregates with ASD (Wang et al., 2014) and unique inherited and *de novo* Reelin variants have been uncovered by exome sequencing of ASD patients (Bonora et al., 2003; Neale et al., 2012; Koshimizu et al., 2013; De Rubeis et al., 2014; Yuen et al., 2015; Zhang et al., 2015). Further evidence for a role of RELN in the etiology of ASD are the reduction of Reelin levels in the cerebellum, frontal cortices and blood of ASD patients (Fatemi, 2001; Fatemi et al., 2005); a reduction of *RELN* and *DAB1* mRNA levels; and an increase in *VLDLR* mRNA levels (Fatemi et al., 2005).

In this study we have characterized the behavior of three zebrafish lines with mutations in Reln signaling pathway components: *reelin*, *dab1a* and *vldlr* (Di Donato et al., 2018). We hypothesized that reduced Reln signaling would decrease social interaction in zebrafish (including shoaling, social preference and aggression) with parallel alterations to monoamine neurotransmitter signaling. Our results demonstrate that *reln^–/–^* mutants display a selective decrease in preference for social novelty that is not observed in *dab1a^–/–^* or *vldlr^–/–^* mutant lines.

## Materials and Methods

### Zebrafish Strains, Care and Maintenance

Zebrafish (*Danio rerio*) were kept at the University of Leicester in accordance with institute animal welfare guidelines. The lighting conditions were 14:10 h (light:dark). Fish were fed twice per day with ZEBRAFEED 400–600 dry food (Sparos). Groups of 15 fish were kept in 3.5 l tanks (Tecniplast). All experiments were approved by a local Animal Welfare and Ethical Review board and were covered by a UK Home Office license to Will Norton. The following strains were used: heterozygous and homozygous *reelin^Δ28–/–^* mutants; *DAB adaptor protein 1a^Δ22–/–^* mutants; *very low density lipoprotein receptor^+13–/–^* mutants (Di Donato et al., 2018); and AB wild-type (WT) zebrafish.

### Behavioral Methods

Behavior was recorded using FlyCapture2 2.5.2.3 software and a digital camera (Point Grey Research). All behavioral experiments were carried out between 11:00 and 17:00. Experiments were performed in a dedicated room with light and temperature kept constant. Zebrafish were moved to the behavior room in holding tanks on the same day as the analysis. Fish were allowed to habituate to the testing room for 1 h. Previous research in our laboratory has demonstrated no sex differences in behavior in our recording setups (e.g., Norton et al., 2011). We therefore used mixed groups of male and female adult zebrafish (3–6 months old) in our analyses. The age and size of fish were carefully matched between genotypes. The sample size (n) for each animal group was calculated based upon power analysis of previous behavioral experiments carried out in our laboratory (e.g., Norton et al., 2011, 2019; Carreno Gutierrez et al., 2017). We only used one experimental setup for each behavioral test, but we cleaned the setup between recordings and changed the water in the tank for each fish tested. Ethovision XT (Noldus) software was used for video tracking. Each film was analyzed by two researchers blind to the genotype or treatment. This removes observer bias in manual quantification.

#### Novel Tank Diving Test

Anxiety-like behavior and exploratory activity was recorded in the novel tank test (NTT) which was performed in a 1.5 L trapezoid tank (Egan et al., 2009). Fish were recorded in this setup for 5 min. We analyzed the time spent in the bottom (geotaxis) and the total distance swum. For *reln*: *n* = 13 WT, *n* = 13 *reln*^+/–^, *n* = 12 *reln*^–/–^. For *dab1a*: *n* = 10 WT, *n* = 7 *dab1a*^–/–^. For *vldlr*: *n* = 7 WT, *n* = 7 *vldlr*^–/–^.

#### Open Field Test

The open field test was carried out in a open tank (40 × 25 cm) filled with water to a depth of 8 cm. Single adult zebrafish were filmed from above for 5 min. The total distance swum, duration of thigmotaxis (time spent at a distance of 2 cm or less from the walls) and time in the center of the tank (representing half of the total tank area) were quantified. For *reln*: *n* = 12 WT, *n* = 12 *reln*^+/–^, *n* = 12 *reln*^–/–^. For *dab1a*: *n* = 10 WT, *n* = 7 *dab1a*^–/–^. For *vldlr*: *n* = 8 WT, *n* = 7 *vldlr*^–/–^.

#### Shoaling Test

Shoaling was recorded in tanks measuring 43 × 22 cm filled with water to a depth of 8 cm. Groups of five familiar adult fish were introduced to the tank, allowed to acclimatize and recorded from above for 10 min (Parker et al., 2013). A mix of males and females were examined. We tracked the fish and measured the average inter-individual distance, polarization and speed of locomotion using VpCore2 software (ViewPoint Life Sciences). *n* = 2 groups of 5 WT, *n* = 2 groups of 5 *reln*^+/–^ and *n* = 2 groups of 5 *reln*^–/–^.

#### Social Preference Test

The social preference test was carried out as described in Carreno Gutierrez et al. (2019). We used a transparent plastic tank containing five compartments: a central area (13 × 19 cm) with two small 6.5 × 9 cm areas either side. The walls separating the central and the side compartments contained holes (1 mm) to allow movement of water and odorants. A single fish was introduced into the central area and permitted to interact with a group of three fish placed in one of the side compartments. To analyze the interactions the central arena was divided in four equal sections and the time spent by the focal fish in each area was recorded. Social preference. In the first session (interaction 1) a group of three unfamiliar WT fish (1st strangers) were placed into one of the side compartments. The behavior of the focal fish was recorded for 5 min, and the time spent closest to the 1st group of strangers was compared to the time spent near the empty area diagonally opposite. Preference for social novelty. A second group of three unfamiliar WT fish (2nd strangers) were placed in the compartment diagonally opposite the first group. The focal fish was recorded for a further 5 min. The time in the area nearest the 1st strangers and the time spent in the quadrant nearest the 2nd strangers was compared. We used a mixture of size-matched males and females as stimuli since can attract both male and female zebrafish (Ruhl et al., 2009). The stimulus fish were changed after recording three focal fish, to standardize the stimulus used and reduce the amount of stress that each animal was exposed to. For *reln*: *n* = 12 WT, *n* = 12 *reln*^+/–^, *n* = 10 *reln*^–/–^. For *dab1a*: *n* = 10 WT, *n* = 7 *dab1a*^–/–^. For *vldlr*: *n* = 8 WT, *n* = 7 *vldlr*^–/–^.

#### Aggression

Aggression was measured using a mirror as a stimulus (Norton et al., 2011). Fish were recorded for 5 min from above. The time spent being aggressive (biting the mirror image and thrashing the tail fin) was quantified manually using LabWatcher (ViewPoint Life Sciences). Films were renamed so that the observer was blind to the genotype being analyzed. For *reln*: *n* = 12 WT, *n* = 12 *reln*^+/–^, *n* = 8 *reln*^–/–^. For *dab1a*: *n* = 10 WT, *n* = 7 *dab1a*^–/–^. For *vldlr*: *n* = 8 WT, *n* = 7 *vldlr*^–/–^.

### Drug Administration

Buspirone hydrochloride was bought from Tocris (Cat. no. 0962), oxytocin was purchased from Sigma Aldrich (Cat. no. O3251) and risperidone was purchased from Tocris (Cat. no. 2865). Administration and concentration of the treatments were chosen according to previously published studies. Buspirone was applied by immersion in water containing 10 mg/L drug for 1 h (Bencan et al., 2009). Oxytocin was applied at a concentration of 10 ng/kg by intraperitoneal injection 30 min before recording (Zimmermann et al., 2016). Risperidone was applied by immersion in water containing 170 μg/L drug for 15 min (Idalencio et al., 2015). For buspirone: *n* = 8 WT, *n* = 8 *reln*^–/–^. For oxytocin: *n* = 9 WT, *n* = 9 *reln*^–/–^. For risperidone: *n* = 9 WT, *n* = 9 *reln*^–/–^.

### Reelin Immunohistochemistry

The anti-Reelin antibody was purchased from Millipore (Cat. no. MAB5366). Immunohistochemistry labeling was carried out using the following protocol. Dissected brains were fixed in 4% PFA for 24 h at 4°C. Brains were washed in phosphate buffered saline (PBS) and stored in methanol at −20°C until processing. Coronal and sagittal sections (100 μM) were cut using a Leica VT1000 S vibratome (Leica Biosystems). After blocking in PBS with 5% normal goat serum (Sigma Cat. no. G9023), 1% dimethyl sulphoxide (Sigma, Cat. no. 276855) and 0.2% Triton X-100 (Fisher, Cat. no. 10254640), sections were incubated in primary antibody for 24 h at 4°C. The secondary antibody (Biotinylated Universal Antibody anti-mouse and rabbit IgG (H + L); Vector Laboratories Cat. no. BA-1400) was incubated for 2 h at room temperature and staining was developed using diaminobenzidine (DAB). Stained sections were mounted and then photographed using an optical microscope (GXM L3200B, GT Vision) and images were assembled in Adobe Photoshop version CS2 (Adobe systems).

### High Pressure Liquid Chromatography (HPLC) Analysis of Monoamines and Their Metabolites

HPLC was performed as described in Carreno Gutierrez et al. (2017). Fish were sacrificed using a schedule 1 procedure. Dissected brains were divided into telencephalon, diencephalon, optic tectum and hindbrain. Samples were prepared in 100 μl ice-cold 0.1 N perchloric acid and centrifuged. HPLC with electrochemical detection was used to measure dopamine (DA), serotonin (5-HT), 3,4-dihydroxyphenylacetic acid (DOPAC), homovanillic acid (HVA) and 5-hydroxyindoleacetic acid (5-HIAA). Samples were compared to standard neurotransmitter solutions and the results were expressed as pmol/mg of brain. 8 *reln^–/–^* and 8 AB brains were processed for HPLC.

### Data Analysis

All data were stored in Excel (Microsoft). Statistical analysis was performed in GraphPad Prism7. Bars represent average values and error bars denote standard error of the mean (SEM). The distribution of the data was verified before choosing an appropriate statistical test, using either the D’Agostino-Pearson or Shapiro–Wilk normality test. A Student’s *t*-test (with Holm–Sidak correction) or Mann–Whitney *U*-test (with Welch correction if appropriate) was used to compare two data sets. When comparing three groups, a one-way ANOVA followed by a Dunnett’s *post hoc* test was used. For non-parametric data a Kruskal–Wallis test followed by Dunn *post hoc* was used. Three-way ANOVA followed by Sidak’s *post hoc* test for multiple comparisons was used to analyze the drug treatment experiments, and two-way ANOVA for the social interaction tests as described in the figure legends. The Statistical significance was shown as follows: ^∗^*p* < 0.05, ^∗∗^*p* < 0.01, ^∗∗∗^*p* < 0.001, ^****^p < 0.0001. The number of animals used is denoted by n in the figure legends.

## Results

### Localization of Reln in the Adult Zebrafish Brain

**FIGURE 1:**
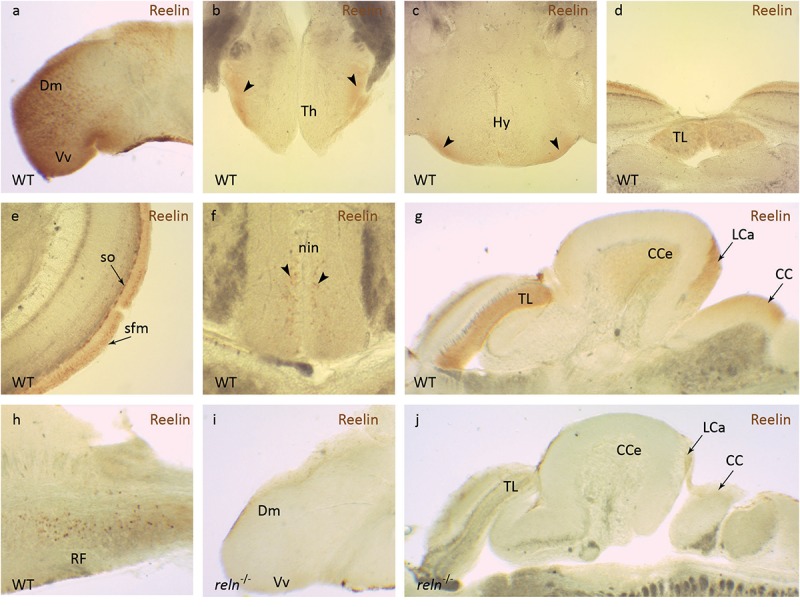
Reelin immunohistochemistry. Anti-Reelin antibody labeling of coronal and sagittal sections of the adult zebrafish brain. In wild-type anti-Reelin antibody labeling is seen in the **(a)** medial part of the dorsal telencephalon (Dm) and in the ventral nucleus of the ventral telencephalon (Vm), in the **(b)** ventral thalamic nuclei (Th) and **(c)** the ventral hypothalamic nuclei (Hy). Labeling is also detected in the **(d)** torus longitudinalis (TL), **(e)** stratum fibrosum marginale (sfm) and the stratum opticum (so) of the optic tectum. **(f)** A few labeled cells are observed in the interpeduncular nucleus (nin). In the hindbrain, Reelin protein localizes to the **(g)** corpus cerebelli (CCe), the lobus caudalis cerebelli (LCa), crista cerebellaris (CC) and **(h)** the intermediate and inferior part of the reticular formation (RF). **(i,j)** Anti-Reelin antibody labeling is not detected in the brain of reln^–/–^ mutants. Black arrowheads show position of Reelin-positive areas in **(b,c,f)**.

We first examined the location of Reelin (Reln) protein in the adult zebrafish brain. In agreement with other published studies, Reln was expressed in a restricted pattern in the adult zebrafish brain, including areas that are important for synaptic organization and plasticity (Costagli et al., 2002). In WT, Reln protein is detected in the medial part of the dorsal telencephalon (Dm) and the ventral nucleus of the ventral telencephalon (Vv, Figure 1a). In the diencephalon, Reelin is found in the ventral thalamic nuclei (Th, Figure 1b) and in the ventral hypothalamic nuclei (Hy, Figure 1c). In the midbrain Reelin is detected in the torus longitudinalis (TL, Figure 1d) as well as the stratum fibrosum marginale (sfm) and stratum opticum (so, Figure 1e) layers of the tectal neuropil. A few labeled cells were observed in the interpeduncular nucleus (nin, Figure 1f). In the hindbrain Reelin is localized to three areas of the cerebellum: the granular cell layer of the corpus cerebelli (CCe), the caudal lobe (LCa) and the crista cerebellaris (CC, Figure 1g). No Reelin expression is observed in the Purkinje and molecular cell layer. Scattered nuclei expressing Reelin are also seen in the intermediate and inferior part of the reticula formation (RF) of the medulla oblongata (Figure 1h). As expected, there is a strong reduction in the level of Reln protein detected in *reln*^–/–^ mutant zebrafish (Figures 1i,j), confirming the specificity of our immunohistochemical analysis.

### Reduction of *reln* Alters Social Behavior

**FIGURE 2:**
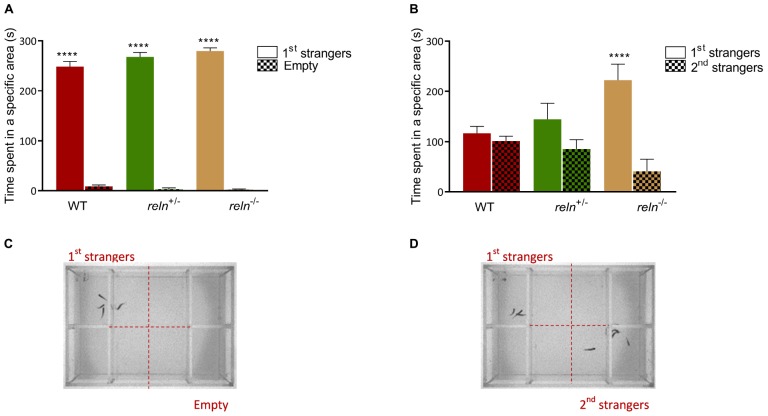
Social preference test. **(A)** Social preference. WT, reln^+/–^ and reln^–/–^ show a significant preference to spend time near a group of unfamiliar fish [1st strangers; *p* < 0.0001 for all genotypes; two-way ANOVA followed by Sidak’s *post hoc*, genotype factor: *F*(2,62) = 1.818, *p* = 0.17, stranger factor: *F*(1,62) = 2392, *p* < 0.0001, interaction genotype × stranger: *F*(2,62) = 4.418, *p* = 0.02]. **(B)** Preference for social novelty. Both WT and *reln*^+/–^ spend an equal amount of time near both groups of unfamiliar fish (1st and 2nd strangers; *p* = 0.99 for WT and p = 0.38 for *reln*^+/–^). *reln^–/–^* do not switch preference to the second group of unfamiliar fish [2nd strangers; *p* < 0.0001; two-way ANOVA followed by Sidak’s *post hoc*, genotype factor: *F*(2,62) = 0.5184, *p* = 0.60, stranger factor: *F*(1,62) = 21.49, p < 0.0001, interaction genotype × stranger: *F*(2,62) = 6.886, *p* = 0.002]. *n* = 12 wild-type, *n* = 12 *reln*^+/–^ and *n* = 10 *reln^–/–^*. ^****^p < 0.0001. Mean ± SEM. **(C,D)** Photographs showing the experimental setup for social preference **(C)** and preference for social novelty **(D)**.

**FIGURE 3:**
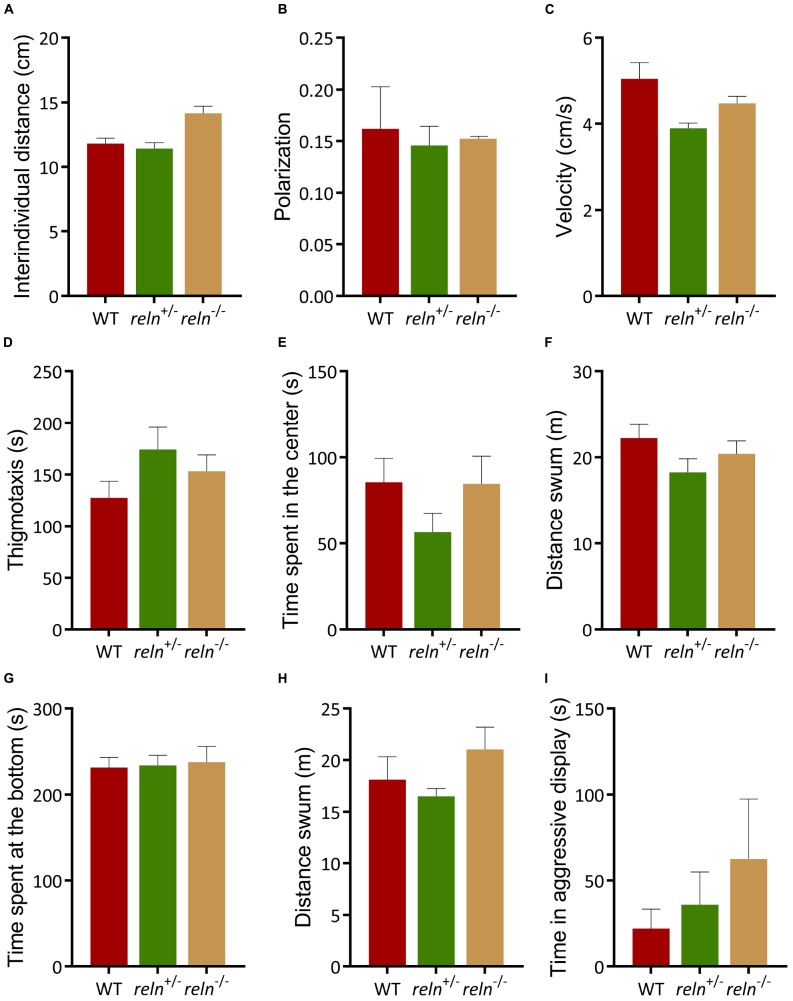
Behavior of *reln*^+/–^ and *reln^–/–^* zebrafish. **(A–C)** Both genotypes shoal normally. **(A)** Inter-individual distance (Kruskal–Wallis test with Dunn’s multiple comparisons test: chi-square = 3.71, *p* > 0.99 for *reln*^+/–^ and *p* = 0.36 for *reln^–/^*^–^); **(B)** Polarization (Kruskal–Wallis test with Dunn’s multiple comparisons (test: chi-square = 0, *p* > 0.99 for both genotypes) and **(C)** velocity [one-way ANOVA Dunnett’s multiple comparisons test: *F*(2,3) = 5.498, *p* = 0.09 for WT, *p* = 0.31 for *reln*^+/–^ and *p* = 0.07 for *reln^–/^*^–^]. *n* = 2 groups of 5 WT, *n* = 2 groups of 5 *reln*^+/–^ and *n* = 2 groups of 5 *reln^–/–^*. **(D–F)** Both *reln*^+/–^ and *reln^–/–^* behave similarly to WT in the open field test. **(D)** Time at the side of the tank [thigmotaxis, one-way ANOVA Dunnett’s multiple comparisons test: *F*(2,33) = 1,687, *p* = 0.20, *p* = 0.13 for WT vs. *reln*^+/–^ and *p* = 0.50 for WT vs. *reln^–/–^*]; **(E)** time spent in the center of the tank [one-way ANOVA Dunnett’s multiple comparisons test: *F*(2,33) = 1,431, *p* = 0.25 for WT, *p* = 0.25 for *reln*^+/–^ and *p* = 0.99 for *reln^–/–^*]. **(F)** Locomotion is not affected in the open field test [one-way ANOVA Dunnett’s multiple comparisons test: *F*(2,33) = 1,625, *p* = 0.21, *p* = 0.14 for *reln*^+/–^ and *p* = 0.61 for *reln^–/–^*]. *n* = 12 WT, *n* = 12 *reln*^+/–^, *n* = 12 *reln^–/–^*. **(G,H)** Both genotypes exhibit normal anxiety-like behavior. **(G)** Time at the bottom of a novel tank (Kruskal–Wallis test with Dunn’s multiple comparisons test: chi-square = 0,5705, *p* = 0.93 for *reln*^+/–^ and *p* > 0.99 for *reln^–/–^*); **(H)** locomotion in a novel tank [one-way ANOVA Dunnett’s multiple comparisons test: *F*(2,35) = 1,54, *p* = 0.23 for WT, *p* = 0.75 for *reln*^+/–^ and *p* = 0.43 for *reln^–/^*^–^) *n* = 13 wild-type, *n* = 13 reln^+/–^, *n* = 12 *reln^–/–^*. **(I)** No difference in aggression levels between WT, *reln*^+/–^ and *reln^–/–^* (Kruskal–Wallis test with Dunn’s multiple comparisons test: chi-square = 3,372, *p* = 0.97 for *reln*^+/–^ and *p* = 0.13 for *reln^–/–^*) *n* = 12 wild-type, *n* = 12 *reln*^+/–^, *n* = 8 *reln*^–/–^. Mean ± SEM.)

The well-established links between Reelin and social behavior prompted us to measure shoaling and social preference in *reln* mutants. We examined social interaction and discrimination in the social preference. We introduced a focal WT or mutant fish in the central compartment of the social preference tank and measured its interaction with a group of unfamiliar WT stimulus fish (1st strangers) (Figure 2C). WT, *reln*^+/–^ and *reln^–/–^* spent more time next to the strangers than in the empty control area [Figure 2A; *p* < 0.0001 for all genotypes; two-way ANOVA followed by Sidak’s *post hoc*, genotype factor: *F*(2,62) = 1.818, *p* = 0.17, stranger factor: *F*(1,62) = 2392, *p* < 0.0001, interaction genotype × stranger: *F*(2,62) = 4.418, *p* = 0.02]. We assessed social novelty preference by placing a group of unfamiliar fish (2nd strangers) into the setup (Figure 2D). Although both WT and *reln*^+/–^ changed their preference and spent an equal amount of time interacting with both groups of fish (Figure 2B; *p* = 0.99 for WT and *p* = 0.38 for reln^+/–^), *reln^–/–^* mutants failed to switch preference and remained with the first group of strangers [Figure 2B; *p* < 0.0001; two-way ANOVA followed by Sidak’s *post hoc*, genotype factor: *F*(2,62) = 0.5184, *p* = 0.60, stranger factor: *F*(1,62) = 21.49, *p* < 0.0001, interaction genotype × stranger: *F*(2,62) = 6.886, *p* = 0.002]. We next recorded shoaling in groups of WT or mutants. We placed 5 WT or *reln* mutants into a large tank and measured the inter-individual distance. In contrast to the social preference test, *reln^–/–^* displayed a similar inter-individual distance (Kruskal–Wallis test with Dunn’s multiple comparisons test: chi-square = 3.71, *p* = 0.36), polarization (Kruskal–Wallis test with Dunn’s multiple comparisons test: chi-square = 0, *p* > 0.99) and velocity [one-way ANOVA Dunnett’s multiple comparisons test: *F*(2,3) = 5.498, *p* = 0.07) as WT when interacting with a group of conspecifics (Figures 3A–C)]. Taken together, these results suggest that *reln^–/–^* mutants display a selective reduction in their preference for social novelty in the absence of global changes to social interactions.

### Exploration, Anxiety-Like Behavior and Aggression Are Not Altered in *reln^–/–^*

We examined the selectivity of this behavioral phenotype by measuring changes to exploration, anxiety-like behavior and aggression in *reln^–/–^* mutant zebrafish. In the open field test, both WT and *reln^–/–^* spent a similar amount of time at the side [Figure 3D; thigmotaxis, *p* = 0.13 for WT vs. *reln*^+/–^ and *p* = 0.50 for WT vs. *reln^–/–^*; one-way ANOVA with Dunnett’s multiple comparisons test: *F*(2,33) = 1.687, *p* = 0.20] and in the center of the tank [Figure 3E; *p* = 0.25 for *reln*^+/–^ and *p* = 0.99 for *reln^–/–^*; one-way ANOVA with Dunnett’s multiple comparisons test: *F*(2,33) = 1.431, *p* = 0.25]. They also swam a similar distance suggesting that exploration is not altered in mutant fish [Figure 3F; *p* = 0.14 for *reln*^+/–^ and *p* = 0.61 for *reln^–/–^*; one-way ANOVA with Dunnett’s multiple comparisons test: *F*(2,33) = 1.625, *p* = 0.21]. *reln^–/–^* also exhibited normal anxiety-like behavior in the NTT. They spent a similar amount of time at the bottom of the tank (Figure 3G; Kruskal–Wallis test with Dunn’s multiple comparisons test: chi-square = 0.5705, *p* = 0.93 for *reln*^+/–^ and *p* > 0.99 for *reln^–/–^*) and swam a similar distance [Figure 3H; *p* = 0.75 for *reln*^+/–^ and *p* = 0.43 for *reln^–/–^*; one-way ANOVA with Dunnett’s multiple comparisons test: *F*(2,35) = 1.54, *p* = 0.23 as WT]. Both genotypes also spent a similar amount of time being aggressive in a mirror test, quantified as the amount of time spent biting the mirror image and thrashing the caudal fin (Figure 3I; Kruskal–Wallis test with Dunn’s multiple comparisons test: chi-square = 3,372, *p* = 0.97 for *reln*^+/–^ and *p* = 0.13 for *reln^–/–^*). Taken together, these results suggest that *reln^–/–^* mutants exhibit a selective reduction in the reaction to social novelty.

### Reaction to Social Novelty Is Not Mediated by the Canonical Reelin Signaling Pathway

**FIGURE 4:**
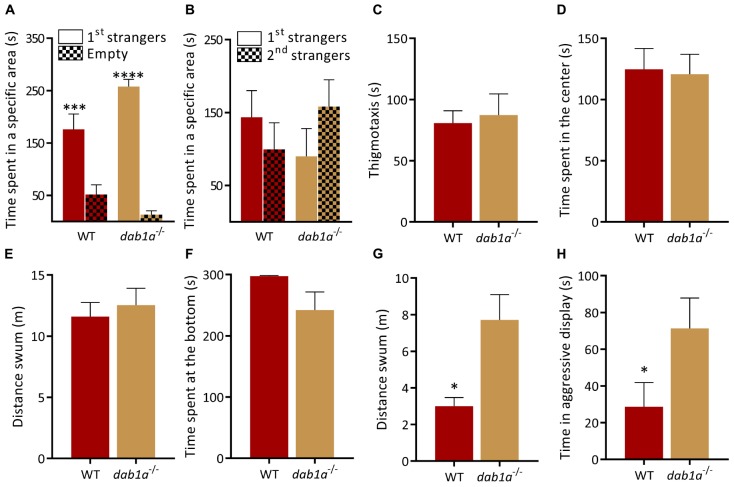
Behavior of *dab1a^–/–^* zebrafish. **(A,B)** Social preference test. **(A)** Both genotypes prefer to spend time near a group of unfamiliar fish [1st strangers; *p* = 0.0005 and *p* < 0.0001 respectively; two-way ANOVA followed by Sidak’s *post hoc*, genotype factor: *F*(1,30) = 0.9841, *p* = 0.3291, stranger factor: *F*(1,30) = 72.81, *p* < 0.0001, interaction genotype × stranger: *F*(1,30) = 7.716, *p* = 0.0093]. **(B)** WT and *dab1a^–/–^* spend equal time near both groups when a second unfamiliar group is added [*p* = 0.61 for WT and *p* = 0.44 for *dab1a^–/–^*; two-way ANOVA followed by Sidak’s *post hoc*, genotype factor: *F*(1,30) = 0.004, *p* = 0.95, stranger factor: *F*(1,30) = 0.1016, *p* = 0.75, interaction genotype × stranger: *F*(1,30) = 2.175, *p* = 0.15. *n* = 10 wild-type and *n* = 7 *dab1a*^–/–^]. **(C–E)** *dab1a*^–/–^ exhibit normal behavior in the open field test. **(C)** Time at side of the tank [thigmotaxis, *t*-test (Welch): *t*_(0__.3262__)_ = 9.971, *p* = 0.75] and **(D)** time in center of the tank [*t*-test (Welch): *t*_(0__.1684__)_ = 14.64, *p* = 0.87] **(E)** locomotion [*t*-test (Welch): *t*_(0__.5277__)_ = 13.05, *p* = 0.61. *n* = 10 wild-type and *n* = 7 *dab1a^–/–^*]. **(F)** *dab1a^–/–^* exhibit normal anxiety-like behavior. Time at the bottom of novel tank (Mann–Whitney test: *U* = 17, *p* = 0.06). **(G)** *dab1a^–/–^* fish are more active than WT in the NTT [*t*-test (Welch): *t*_(3__.234__)_ = 7.409, *p* = 0.0133. *n* = 10 wild-type and *n* = 7 *dab1a^–/–^*]. **(H)** *dab1a^–/–^* display heightened aggression levels compared to WT (Mann–Whitney test: *U* = 11.5, *p* = 0.0445. *n* = 10 wild-type and *n* = 7 *dab1a^–/–^*). ^∗^*p* < 0.05, ^∗∗∗^*p* < 0.001, ^****^p < 0.0001. Mean ± SEM.

Reelin activity is transduced by several downstream pathways including canonical signaling via DAB adaptor protein 1 and the Very low density lipoprotein receptor (Vldlr) (Trommsdorff et al., 1999; Bock and May, 2016). We characterized *dab1a^–/–^* (one of the zebrafish homologs of mammalian *Dab1*) and *vldlr^–/–^* mutant lines to examine the contribution of canonical signaling to the phenotype of *reln^–/–^* zebrafish. Interestingly, both *dab1a^–/–^* and *vldlr^–/–^* displayed different behavioral profiles. In the social preference test, *dab1a^–/–^* showed a similar social preference [Figure 4A; WT, *p* = 0.0005 and *dab1a^–/–^*, *p* < 0.0001; two-way ANOVA followed by Sidak’s *post hoc*, genotype factor: *F*(1,30) = 0.9841, *p* = 0.3291, stranger factor: *F*(1,30) = 72.81 *p* < 0.0001, interaction genotype × stranger: *F*(1,30) = 7.716 *p* = 0.0093] and reaction to social novelty as WT [Figure 4B; *p* = 0.61 for WT and *p* = 0.44 for *dab1a^–/–^*; two-way ANOVA followed by Sidak’s *post hoc*, genotype factor: *F*(1,30) = 0.004, *p* = 0.95, stranger factor: *F*(1,30) = 0.1016, *p* = 0.75, interaction genotype × stranger: *F*(1,30) = 2.175, *p* = 0.15]. Exploration in the open field test was similar in *dab1a^–/–^* and WT [Figures 4C–E; thigmotaxis, *t*-test (Welch): *t*_(__0__.__3262__)_ = 9.971, *p* = 0.75; time in center of the tank, *t*-test (Welch): *t*_(__0__.__1684__)_ = 14.64, *p* = 0.87 and locomotion, *t*-test (Welch): *t*_(__0__.__5277__)_ = 13.05, *p* = 0.61]. Both genotypes also spent a similar amount of time at the bottom of a novel tank (Figure 4F; Mann–Whitney test: *U* = *17*, *p* = 0.06), a readout of anxiety-like behavior, whereas mutants were hyperactive compared to WT [Figure 4G; *t*-test (Welch): *t*_(__3__.__234__)_ = 7.409, *p* = 0.01]. Finally, *dab1a^–/–^* mutants were more aggressive than WT in the mirror aggression test (Figure 4H; Mann–Whitney test: *U* = 11.5, *p* = 0.0445).

**FIGURE 5:**
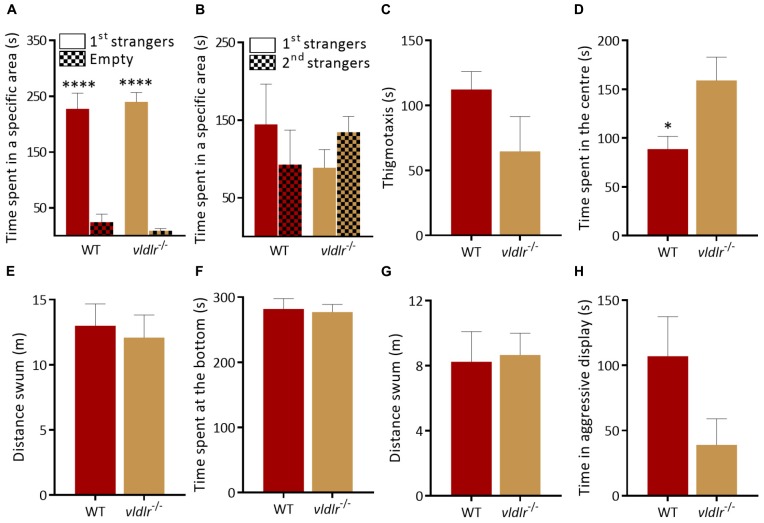
Behavior of *vldlr^–/–^* zebrafish. **(A,B)** Social preference test. **(A)** Both genotypes prefer to spend time near a group of unfamiliar fish [1st strangers; *p* < 0.0001 for both genotypes; two-way ANOVA followed by Sidak’s *post hoc*, genotype factor: *F*(1,24) = 0.0039, *p* = 0.95, stranger factor: *F*(1,24) = 120.1, *p* < 0.0001, interaction genotype × stranger: *F*(1,24) = 0.4912, *p* = 0.49]. **(B)** WT and *vldlr*^–/–^ spend equal time near both groups when a second unfamiliar group is added [*p* = 0.58 for WT and *p* = 0.73 for *vldlr^–/–^;* two-way ANOVA followed by Sidak’s *post hoc*, genotype factor: *F*(1,24) = 1.353, *p* = 0.26, stranger factor: *F*(1,24) = 0.005, *p* = 0.94, interaction genotype × stranger: *F*(1,24) = 0.02779, *p* = 0.87. *n* = 8 wild-type and *n* = 7 *vldlr^–/–^*]. **(C–E)** *vldlr^–/–^* behavior in the open field test. **(C)** *vldlr^–/–^* spent the same time at side of the tank [*t*-test (Welch): *t*_(1__.581__)_ = 9.068, *p* = 0.15] compared to WT but more **(D)** time in center of the tank [*t*-test (Welch): *t*_(2__.595__)_ = 9.47, *p* = 0.03]. **(E)** Locomotion [*t*-test (Welch): *t*_(0__.3779__)_ = 12.84, *p* = 0.71. *n* = 8 wild-type and *n* = 7 *vldlr^–/–^*]. **(F)** *vldlr^–/–^* exhibit normal anxiety-like behavior. Time at the bottom of novel tank (Mann Whitney test: *U* = 16, *p* = 0.32), **(G)** locomotion in a novel tank [*t*-test (Welch): *t*_(0__.1803__)_ = 10.92, *p* = 0.86. *n* = 7 wild-type and *n* = 7 *vldlr*^–/–^]. **(H)** No difference in aggression levels between WT and *vldlr^–/^*^–^ [*t*-test (Welch): *t*_(1__.87__)_ = 11.41, *p* = 0.09. *n* = 8 WT and *n* = 7 *vldlr^–/–^*]. ^∗^*p* < 0.05, ^****^p < 0.0001.

*vldlr^–/–^* mutants also showed both a similar interaction with the first group of strangers [Figure 5A; *p* < 0.0001 for both genotypes; two-way ANOVA followed by Sidak’s *post hoc*, genotype factor: *F*(1,24) = 0.0039, *p* = 0.95, stranger factor: *F*(1,24) = 120.1, *p* < 0.0001, interaction genotype × stranger: *F*(1,24) = 0.4912, *p* = 0.49] and a normal reaction to social novelty [Figure 5B; *p* = 0.58 for WT and *p* = 0.73 for *vldlr^–/–^*; two-way ANOVA followed by Sidak’s *post hoc*, genotype factor: *F*(1,24) = 1.353, *p* = 0.26, stranger factor: *F*(1,24) = 0.005, *p* = 0.94, interaction genotype × stranger: *F*(1,24) = 0.02779, *p* = 0.87] as WT in the social preference test. In the open field test, *vldlr*^–/–^ spent the same time at side of the tank [Figure 5C; *t*-test (Welch): *t*(_1.581_) = 9.068, *p* = 0.15] compared to WT but *vldlr^–/–^* mutants spent more significantly more time in the center of an open field tank than WT [Figure 5D; *t*-test (Welch): *t*_(2.595)_ = 9.47, *p* = 0.03]. However, the distance swum in the open field was the same as WT [Figure 5E; *t*-test (Welch): *t*_(__0__.__3779__)_ = 12.84, *p* = 0.71] suggesting that *vldlr^–/–^* are more explorative or less anxious than WT without being hyperactive. Anxiety-like behavior in the NTT was not altered in mutants [Figures 5F,G; time at bottom of tank, Mann–Whitney test: *U* = 16, *p* = 0.32 and locomotion, *t*-test (Welch): *t*_(__0__.__1803__)_ = 10.92, *p* = 0.86]. Finally, mutants also exhibited a trend decrease in aggression levels that did not reach significance [Figure 5H; *t*-test (Welch): *t*_(__1__.__87__)_ = 11.41, *p* = 0.09]. In summary, although mutation of *reln* leads to a specific alteration in social interactions, this phenotype does not appear to be mediated by the canonical Reln signaling components *dab1a* and *vldlr*, which display different behavioral alterations.

### Increased Levels of 5-HT in the Hindbrain of *reln^–/–^*

**FIGURE 6:**
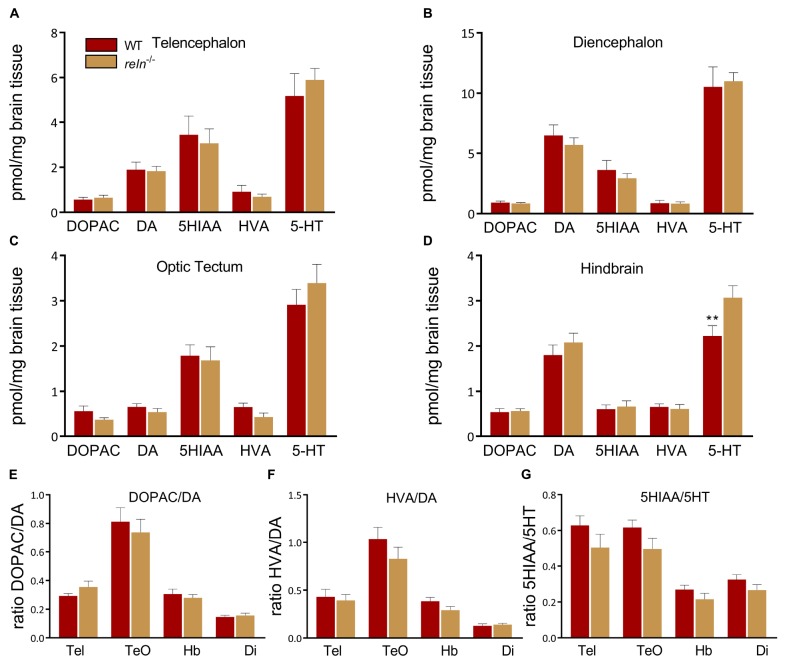
High pressure liquid chromatography. 5-HT levels are increased in the hindbrain of *reln^–/–^* compared to WT [*t*-test: *t*_(3__.694__)_ = 70, *p* = 0.0021, multiple *t*-tests with Holm–Sidak multiple comparisons correction. *n* = 8 WT, *n* = 8 *reln*^–/–^]. **(A)** Telencephalon, **(B)** diencephalon, **(C)** optic tectum and **(D)** hindbrain. No differences in the breakdown of **(E)** DOPAC/DA, **(F)** HVA/DA, **(G)** 5-HIAA/-5HT. DA, dopamine; Di, diencephalon; DOPAC, 3,4-Dihydroxyphenylacetic acid; Hb, Hindbrain; HVA, homovanillic acid; Tel, telencephalon; TeO, optic tectum; 5-HIAA, 5-hydroxyindoleacetic acid; 5-HT, 5-hydroxytryptamine. ^∗∗^*p* < 0.01. Mean ± SEM.

The control of social behavior, aggression and anxiety has been linked to monoamine signaling in the brain (Popova, 2008; Herculano and Maximino, 2014; Carreno Gutierrez et al., 2019). We used high pressure liquid chromatography (HPLC) to quantify the levels of 5-HT, DA and metabolites in WT and *reln* mutant zebrafish. Since heterozygous *reln*^+/–^ mutants did not show behavioral alterations compared to WT we focused on *reln^–/–^* animals in these experiments. Using HPLC we uncovered a selective increase of 5-HT in the hindbrain of *reln^–/–^* compared to WT [Figure 6D; *t*-test: *t*_(__3__.__694__)_ = 70, *p* = 0.0021, multiple *t*-tests with Holm–Sidak multiple comparisons correction]. In contrast, we detected similar levels of dopamine (DA), homovanillic acid (HVA), dihydroxyphenylacetic acid (DOPAC) and 5-hydroxyindoleacetic acid (5HIAA) in the brain of both genotypes (Figures 6A–D). We also calculated the utilization ratio of each neurotransmitter. HPLC measures the sum basal level of analytes in the brain regardless of whether or not have been released at the synapse. Since neurotransmitters are broken down to their metabolites upon release the utilization ratio gives an approximation of neurotransmitter activity (Kilpatrick et al., 1986). The utilization ratio of 5-HT and DA was unaffected in *reln^–/–^* suggesting that release and reuptake of neurotransmitters is unaffected in these mutants (Figures 6E–G).

### 5-HT Modulates the Preference for Social Interaction in Zebrafish

**FIGURE 7:**
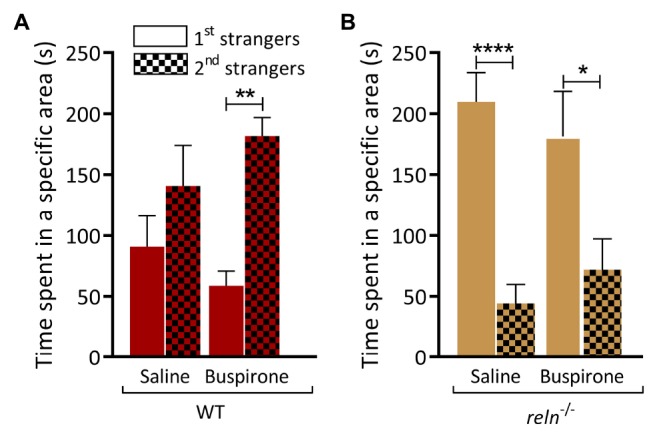
Buspirone increase the social preference for novelty in WT but not *reln^–/–^*. **(A)** WT treated with saline spend equal time near the two groups of unfamiliar fish, whereas WT treated with buspirone switch preference and spend more time near the new group of unfamiliar fish. **(B)** Buspirone treatment does not completely rescue the impaired preference for social novelty of *reln^–/–^* zebrafish (WT with saline *p* = 0.51; WT with buspirone *p* = 0.0039; *reln^–/–^* with saline *p* < 0.0001; *reln^–/–^* with buspirone *p* = 0.012). Three-way ANOVA followed by Sidak’s *post hoc*, genotype factor: *F*(1,56) = 0.2488, *p* = 0.62, stranger factor: *F*(1,56) = 2.11, *p* = 0.16, treatment factor: *F*(1,56) = 0.0124, *p* = 0.91, interaction genotype × stranger: *F*(1,56) = 40.28, *p* < 0.0001, interaction genotype × treatment: *F*(1,56) = 0.01879, *p* = 0.89, interaction stranger × treatment: *F*(1,56) = 3.362, *p* = 0.07, interaction stranger × genotype × treatment: *F*(1,56) = 0.0566, *p* = 0.81). *n* = 8 WT and *n* = 8 *reln^–/–^*. ^∗^*p* < 0.05, ^∗∗^*p* < 0.01, ^****^p < 0.0001. Mean ± SEM.

**FIGURE 8:**
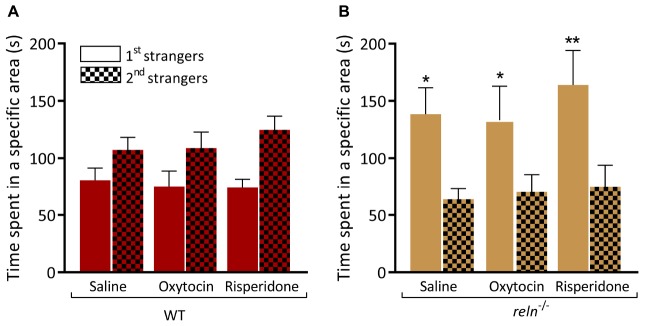
Oxytocin and risperidone do not rescue preference for social novelty. **(A)** WT treated with saline (*p* = 0.57), oxytocin (*p* = 0.53), and risperidone (*p* = 0.14) spend equal time near the two groups of unfamiliar fish. **(B)** Neither oxytocin (*p* = 0.048) nor risperidone (*p* = 0.0023) treatment rescues the impaired preference for social novelty of *reln^–/–^* zebrafish (saline control, *p* = 0.014). Three-way ANOVA followed by Sidak’s *post hoc* for oxytocin, genotype factor: *F*(1,64) = 0.3928, *p* = 0.53, stranger factor: *F*(1,64) = 2.133, *p* = 0.15, treatment factor: *F*(1,64) = 0.0143, *p* = 0.90, interaction genotype × stranger: *F*(1,64) = 17.44, *p* < 0.0001, interaction genotype × treatment: *F*(1,64) = 0.0282, *p* = 0.87, interaction stranger × treatment: *F*(1,64) = 0.0707, *p* = 0.79, interaction stranger × genotype × treatment: *F*(1,64) = 0.04759, *p* = 0.83. Three-way ANOVA followed by Sidak’s *post hoc* for risperidone, genotype factor: *F*(1,64) = 1.073, *p* = 0.30, stranger factor: *F*(1,64) = 2.573, *p* = 0.11, treatment factor: *F*(1,64) = 0.8058, *p* = 0.37, interaction genotype × stranger: *F*(1,64) = 25.65, *p* < 0.0001, interaction genotype × treatment: *F*(1,64) = 0.3363, *p* = 0.56, interaction stranger × treatment: *F*(1,64) = 0.01244, *p* = 0.91, interaction stranger × genotype × treatment: *F*(1,64) = 0.4869, *p* = 0.49. *n* = 9 WT and *n* = 9 *reln^–/–^*. ^∗^*p* < 0.05, ^∗∗^*p* < 0.01. Mean ± SEM.

HPLC analysis identified a significant increase of 5-HT in the hindbrain of *reln^–/–^*. We applied the 5HT-1A receptor agonist buspirone that decreases the concentration of 5-HT at the synapse (Loane and Politis, 2012) and measured behavior. Immersion in buspirone increased the interaction of WT with the second group of unfamiliar fish in the visually mediated social preference test (Figure 7A; first strangers *p* = 0.51 and second strangers *p* = 0.004). However, buspirone did not rescue the mutant phenotype, and *reln^–/–^* still failed to switch social preference following drug application [Figure 7B; second strangers *p* = 0.01; three-way ANOVA followed by Sidak’s *post hoc*, genotype factor: *F*(1,56) = 0.2488, *p* = 0.62, stranger factor: *F*(1,56) = 2.11, *p* = 0.16, treatment factor: *F*(1,56) = 0.0124, *p* = 0.91, interaction genotype × stranger: *F*(1,56) = 40.28, *p* < 0.0001, interaction genotype × treatment: *F*(1,56) = 0.01879, *p* = 0.89, interaction stranger × treatment: *F*(1,56) = 3.362, *p* = 0.07, interaction stranger × genotype × treatment: *F*(1,56) = 0.0566, *p* = 0.81]. In humans, mutations in *RELN* are linked to autism spectrum disorder. We applied the autism treatment drugs oxytocin (Hollander et al., 2003; Andari et al., 2010; Guastella et al., 2010) and risperidone (Canitano and Scandurra, 2008) to *reln^–/–^* mutants and quantified individual social interactions in the social preference test. However, neither oxytocin nor risperidone altered the preference for social novelty in zebrafish of either genotype [Figure 8A; WT saline *p* = 0.57, WT oxytocin *p* = 0.53 and WT risperidone *p* = 0.14, Figure 8B; *reln^–/–^* saline *p* = 0.014, *reln^–/–^* oxytocin *p* = 0.048, *reln^–/–^* risperidone *p* = 0.014; Three-way ANOVA followed by Sidak’s *post hoc* for oxytocin, genotype factor: *F*(1,64) = 0.3928, *p* = 0.53, stranger factor: *F*(1,64) = 2.133, *p* = 0.15, treatment factor: *F*(1,64) = 0.0143, *p* = 0.90, interaction genotype × stranger: *F*(1,64) = 17.44, *p* < 0.0001, interaction genotype × treatment: *F*(1,64) = 0.0282, *p* = 0.87, interaction stranger × treatment: *F*(1,64) = 0.0707, *p* = 0.79, interaction stranger × genotype × treatment: *F*(1,64) = 0.04759, *p* = 0.83. Three-way ANOVA followed by Sidak’s *post hoc* for risperidone, genotype factor: *F*(1,64) = 1.073, *p* = 0.30, stranger factor: *F*(1,64) = 2.573, *p* = 0.11, treatment factor: *F*(1,64) = 0.8058, *p* = 0.37, interaction genotype × stranger: *F*(1,64) = 25.65, *p* < 0.0001, interaction genotype × treatment: *F*(1,64) = 0.3363, *p* = 0.56, interaction stranger × treatment: *F*(1,64) = 0.01244, *p* = 0.91, interaction stranger × genotype × treatment: *F*(1,64) = 0.4869, *p* = 0.49].

## Discussion

In this study we have quantified the contribution of Reelin pathway signaling to adult zebrafish behavior. Zebrafish *reln^–/–^* mutants display a decrease in preference for social novelty, without altering shoaling, anxiety-like behavior, aggression or exploration. Conversely mutants for the canonical Reln targets *dab1a* and *vldlr* display normal social preference, but heightened aggression (*dab1a^–/–^*) and exploration (*vldlr^–/–^*). This suggests that Reln signaling has a pleiotropic role in controlling zebrafish behavior.

### Reelin Localization in the Adult Zebrafish Brain

The zebrafish telencephalon has a non-laminar structure that develops through eversion, a process whereby the dorsal pallial region of the forebrain folds over the more ventral subpallium (Folgueira et al., 2012). Conversely, the mammalian cortex has a six-layered laminar structure that develops through evagination (Nieuwenhuys, 1994; Mueller, 2011). In zebrafish, Reln protein is secreted from superficial inhibitory interneurons (SINs) in a similar manner to the localization of Reln in interneurons in other adult vertebrates (Di Donato et al., 2018). However, Reln could have a different function in the non-laminar zebrafish brain compared to other vertebrates. Antibody labeling of the adult zebrafish brain agrees with the distribution of *reln* mRNA described by Costagli et al. (2002). The optic tectum is the principal retinorecipient brain region in zebrafish and is homologous to the superior colliculus in mammals (Gebhardt et al., 2013). The stratum fibrosum marginale (sfm) is located underneath the pia mater and glia limitans and the stratum opticum (so) is directly below it. The sfm is a plexiform-type layer containing a dense assortment of axons, axon terminals and dendrites, whereas the so is formed of optic nerve terminal bundles (Corbo et al., 2012). Reln is also found in the torus longitudinalis (TL), a ray-finned fish-specific paired longitudinal eminence of granule cells located at the medial margins of the optic tectum. The granule cells of the torus longitudinalis project unmyelinated axons through the contralateral sfm of the optic tectum to synapse upon pyramidal cells. The torus longitudinalis is thought to be involved in learning complex visual scenes (Northmore, 2017). In the isthmic tegmentum, Reln is found in a few cells in the interpeduncular nucleus (nin), a structure that receives projections from the dorsal habenula and may be involved in fear conditioning (Amo et al., 2010). In the hindbrain, granule cells of the corpus cerebelli (CCe) send their axons to Purkinje cells in the molecular layer of the cerebellum, whereas the granule cells of the caudal lobe of the cerebellum (LCa) project to crest cells in the crista cerebellaris (CC; Takeuchi et al., 2015). Therefore, the hindbrain areas that contain Reln are all linked to each other functionally. Scattered Reln-positive cells are also present in the intermediate and inferior part of the reticular formation, an intricate network of excitatory and inhibitory neurons and diffuse nuclei extending from the spinal cord through the medulla oblongata to the mesencephalon.

The presence of Reln in the laminated structures of the optic tectum and cerebellum suggest that this protein may have a conserved role in neuronal migration in some areas of the zebrafish brain. This hypothesis has been confirmed in a study showing synaptic lamination defects in the optic tectum of *reln^–/–^* larvae (Di Donato et al., 2018). Although the optic tectum of adult zebrafish *reln^–/–^* mutants does not show patterning defects, in the cerebellum Purkinje cells, eurydendroid cells and Bergmann glia are found in ectopic positions in the absence of hypoplasia (Nimura et al., 2019). The localization of Reln in the zebrafish brain fits with the idea that this protein could play a role in synaptic organization or the integration of sensory afferent information from tectal or diencephalic brain areas to the dorsal telencephalon (Costagli et al., 2002).

### Loss of Reln Function Decreases Preference for Social Novelty

The presence of RELN at the peak region of linkage and first autism susceptibility locus (chromosome 7q22) suggested that loss of *reln* function might alter zebrafish social behavior. In a shoaling test, groups of mutants displayed similar social interactions as groups of WT zebrafish including inter-individual distance (Figure 3A), polarization (Figure 3B) and velocity (Figure 3C). Furthermore, in a social preference test, *reln^–/–^* mutants interacted normally with a first group of conspecifics (Figure 2A) suggesting that social interactions occur normally in this mutant line. However, *reln^–/–^* fail to interact with a second group of animals placed into the same tank demonstrating a reduction in preference for social novelty (Figure 2B). Other behavioral measures, including exploration of a large tank (Figures 3D–F), anxiety-like behavior (Figures 3G,H), and aggression (Figure 3I) were not altered.

Homozygous *Reln*^–/–^ mutant mice are not suitable for behavioral studies since they display severe ataxia, likely due to their severe neuronal migration defects. However, heterozygous *reeler* mice (*Reln*^+/–^) display behavioral, cognitive and neuroanatomical defects that are milder than those seen in *Reln^–/–^*. RELN haploinsufficiency causes loss of Purkinje neurons in the cerebellum of *Reln*^+/–^ (Biamonte et al., 2009; Maloku et al., 2010), a phenotype that is similar to the loss of Purkinje cells seen in the cerebellum of some ASD patients (Ritvo et al., 1986). Behavioral characterization of *Reln*^+/–^ has provided ambiguous results. Some studies have reported increased anxiety, impaired executive function, motor impulsivity, abnormal prepulse inhibition, decreased contextual fear conditioning and associative learning impairments in *Reln*^+/–^ mice (Tueting et al., 1999; Tremolizzo et al., 2002; Krueger et al., 2006; Qiu et al., 2006) whereas other groups report that *Reln*^+/–^ display normal locomotor activity, prepulse inhibition, anxiety, coordination, social behavior, spatial learning and transitions in the light–dark test, a measure of anxiety (Podhorna and Didriksen, 2004; Qiu et al., 2006). Regarding social interactions and communication, hallmarks of ASD, *Reln*^+/–^ spend more time engaging in social investigation (Podhorna and Didriksen, 2004) but do not display alterations in social and vocal repertoires during courtship (Michetti et al., 2014). However, deletion of the positively charged carboxy-terminal region of RELN attenuates canonical RELN signaling and leads to decreased social interaction, impaired working memory, hyperactivity and decreased anxiety compared to WT (Kohno et al., 2015; Sakai et al., 2016). The surprising range of different behavioral phenotypes that has been reported could be due to the genetic background of the mutant animals, the age of testing or the behavioral protocol used.

### Canonical Reln Signaling Does Not Underpin the Behavioral Phenotype

Reln signals via several downstream pathways including the canonical receptors apolipoprotein E receptor 2 and the very low density lipoprotein receptor (Trommsdorff et al., 1999) both of which lead to Disabled-1 activation (Howell et al., 1997; Sheldon et al., 1997). We investigated the possible contribution of canonical signaling to the phenotype of *reln^–/–^* zebrafish by characterizing the behavior of *dab1a^–/–^* and *vldr^–/–^* mutant lines. Both *dab1a^–/–^* and *vldlr^–/–^* displayed different behavioral profiles. *dab1a^–/–^* fish were hyperactive (Figure 4F) and more aggressive (Figure 4D) compared to WT without showing alterations to social behavior (Figure 4). Conversely, *vldlr^–/–^* exhibited a selective increase in exploration of an open field tank (Figure 5D). Although we did not have access to an *apoer2^–/–^* mutant line, the essential adaptor protein Dab1a represents a common target for both receptors. Therefore, the lack of phenotypic overlap with *reln^–/–^* suggests that we can rule out a contribution of canonical signaling to the decreased preference for social novelty. Studies to address this issue could include examining other Reln signaling targets such as the CrK/Rap1, P13K/Akt/mTOR, and MEK/Erk1/2 pathways (Chen et al., 2010). For example, this could include rescuing the social deficit of *reln^–/–^* mutants by using drugs that stimulate these signaling pathways.

### 5-HT Signaling Contributes to the Decreased Preference for Social Novelty Observed in *reln^–/–^*

Irregularities in several neurotransmitter pathways, including the monoamine 5-HT, have been implicated in the pathology of ASD. We used high pressure liquid chromatography to measure the monoamines dopamine, 5-HT and their metabolites (Figure 6). We observed a significant increase of 5-HT that was restricted to the hindbrain of *reln^–/–^* (Figure 6D), the brain region that contains the raphe nucleus [the largest group of 5-HT producing neurons in zebrafish (Lillesaar et al., 2009) and that expresses the signaling pathways components *tryptophan hydroxylase 2*, *vesicular monoamine transporter* and the 5-HT transporter-encoding gene *solute carrier family 6 member 4a* (Teraoka et al., 2004; Wang et al., 2006; Norton et al., 2008; Wen et al., 2008)]. As well as regulating cell division and differentiation, neurite growth, and synaptogenesis, 5-HT also modulates human behaviors associated with psychiatric disorders (Chugani, 2002). Both hypo- and hyper-serotonemia can occur in ASD patients (Schain and Freedman, 1961; Connors et al., 2006; Yang et al., 2014). We investigated the connection between aberrant 5-HT signaling and social preference by applying the 5-HT_1__A_ receptor partial agonist buspirone (Loane and Politis, 2012). Buspirone acts presynaptically to reduce 5-HT levels (Gebauer et al., 2011) and it can have both anxiolytic and pro-social effects in zebrafish (File and Seth, 2003; Bencan et al., 2009; Gould, 2011; Barba-Escobedo and Gould, 2012; Gould et al., 2012; Maaswinkel et al., 2012; Maaswinkel et al., 2013). Although immersion in buspirone increased the interaction of WT with the second group of unfamiliar fish (Figure 7A) it did not rescue the mutant phenotype, with *reln^–/–^* failing to switch social preference following drug treatment (Figure 7B). This suggests that the 5-HT signaling does not underpin the lack of preference for social novelty seen in *reln^–/–^* zebrafish, and further research is required to understand the neurotransmitter basis of this behavioral phenotype.

## Conclusion

Multiple lines of evidence suggest that RELN is a vulnerability factor for ASD. Characterization of the behavioral phenotype of *reln^–/–^* mutant zebrafish provided an opportunity to investigate which adult zebrafish behaviors can be linked to ASD-candidate genes. Using a combination of behavioral tests we were able to identify a specific alteration in preference for social novelty without a general change to social behavior. It is difficult to compare this finding to other model species such as mouse because manipulation of Reln signaling leads to such a wide range of different phenotypes (Podhorna and Didriksen, 2004; Qiu et al., 2006). The reduction in preference for social novelty that we observed is surprisingly specific and would fit with the definition of ASD that includes restricted interests and repetitive patterns of behavior that are abnormal in intensity or focus. Arguing against this idea, however, we were unable to rescue this phenotype by applying two drugs that are used to treat ASD: oxytocin and risperidone. One explanation for this result could be that we need to apply these drugs for a longer time period or at higher concentration. For example, oxytocin administration to mouse models of ASD symptoms only rescued social deficits behavior 1–2 weeks following sub-chronic application (Teng et al., 2013). Furthermore, since there are extensive connections between oxytocin and 5-HT neurotransmitter signaling co-application of drugs targeting both these pathways may be required to rescue the behavioral phenotype of *reln^–/–^*.

In summary, the abnormal preference for social novelty displayed by *reln*^–/–^ zebrafish mutants suggest that they may represent a good model to analyze some aspects of ASD, including restricted or repetitive behavior. Further research comparing the simultaneous presentation of different types of stimuli in the visually mediated preference test could be used to investigate this idea in more detail. Creation of novel zebrafish *reln* mutant lines that either harbor mutations associated with ASD in human patients or in different genetic backgrounds might provide further insights into the pleiotropic behavioral phenotypes seen in animals lacking Reln signaling.

## Data Availability

The datasets generated for this study are available on request to the corresponding author.

## Ethics Statement

This study was carried out in accordance with the recommendations of the local Animal Welfare and Ethical Review board at the University of Leicester. The protocol was approved by the local Animal Welfare and Ethical Review board at the University of Leicester.

## Author Contributions

ED designed the experiments, collected and analyzed the data, and wrote a first version of the manuscript. VD created the *reln^–/–^*, *dab1a^–/–^* and *vldlr^–/–^* mutant lines. AY helped collect and analyze the HPLC data. FD provided the novel mutant lines. WN analyzed the data and wrote the manuscript. All authors approved the final manuscript before submission.

## Conflict of Interest Statement

The authors declare that the research was conducted in the absence of any commercial or financial relationships that could be construed as a potential conflict of interest.
